# Supplementary material for: TLR9/MyD88/NF-κB signaling mediates mental stress-induced exacerbation of psoriasis through immune dysregulation in a mouse model
Source: PLoS One. 2026 Mar 6;21(3):e0344474. doi: 10.1371/journal.pone.0344474 (PMC12965552; doi:10.1371/journal.pone.0344474)
Supplement: S1 File — (PDF) [file pone.0344474.s001.pdf]

Figure 4A Western blotting for PBMCs isolated from mice

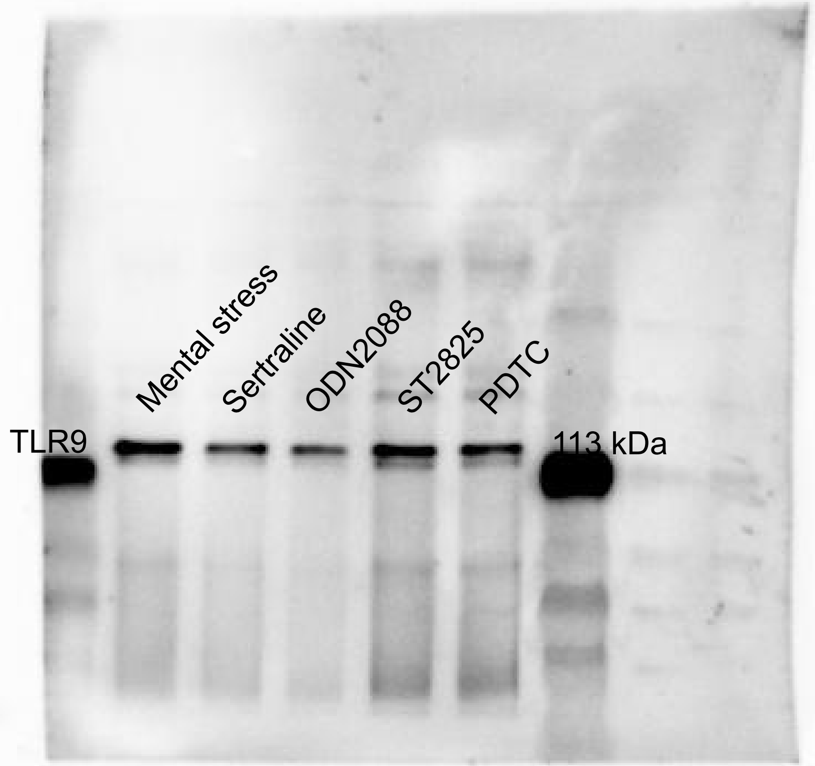
Mental stress
Sertraline
ODN2088
ST2825
PDTC
TLR9
113 kDa

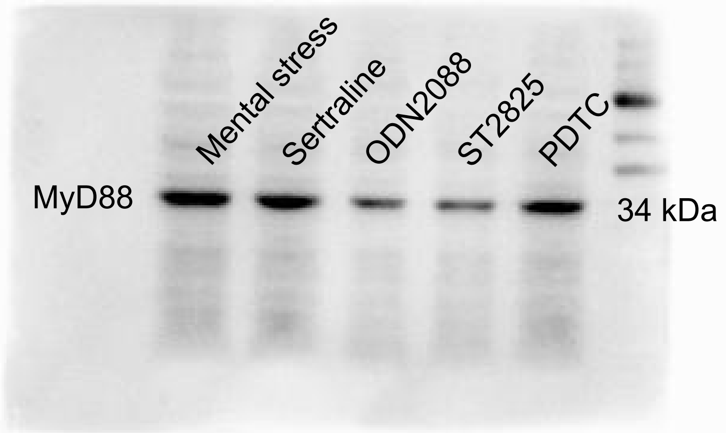
Mental stress
Sertraline
ODN2088
ST2825
PDTC
MyD88
34 kDa

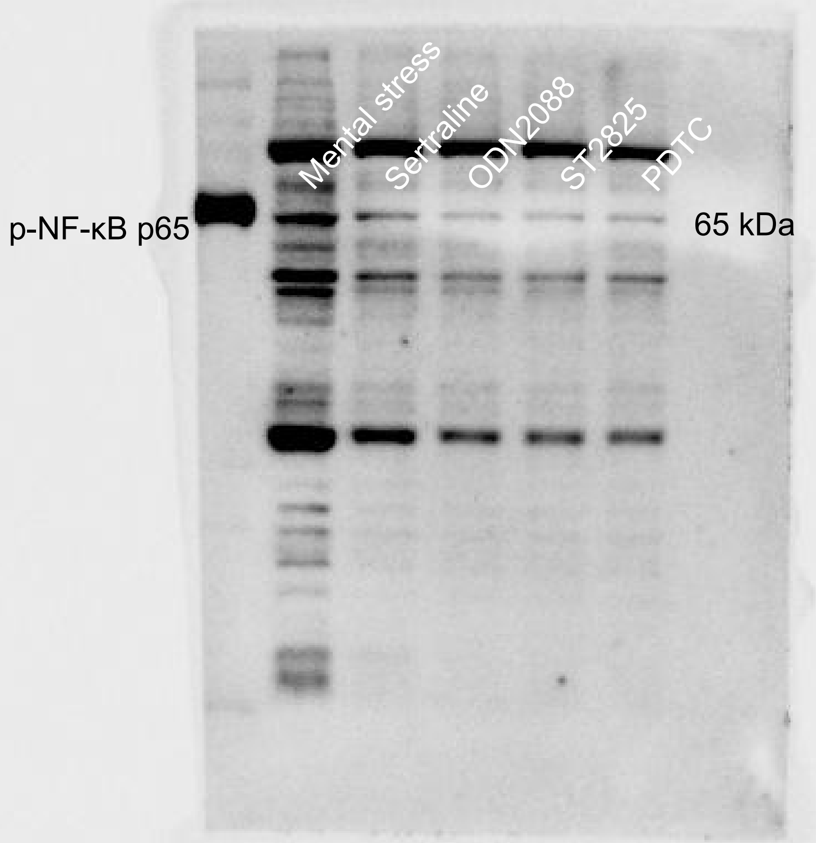
Mental stress
Sertraline
ODN2088
ST2825
PDTC
p-NF-κB p65
65 kDa

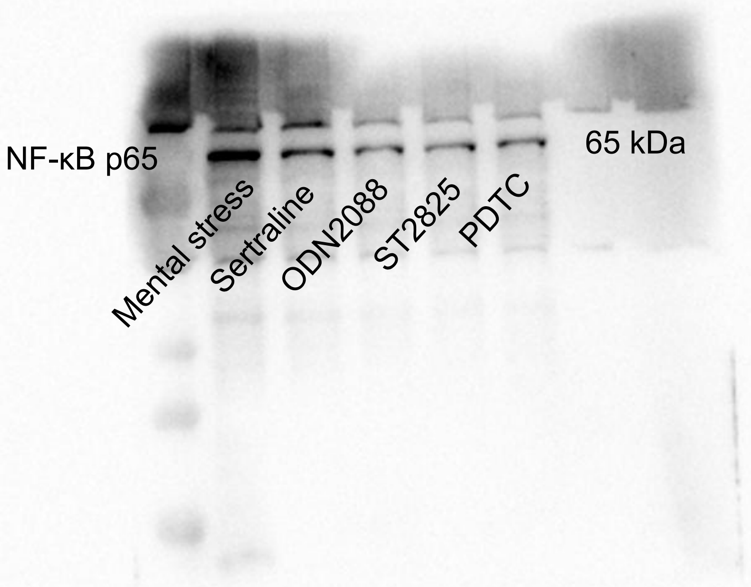
NF-κB p65
Mental stress
Sertraline
ODN2088
ST2825
PDTC
65 kDa

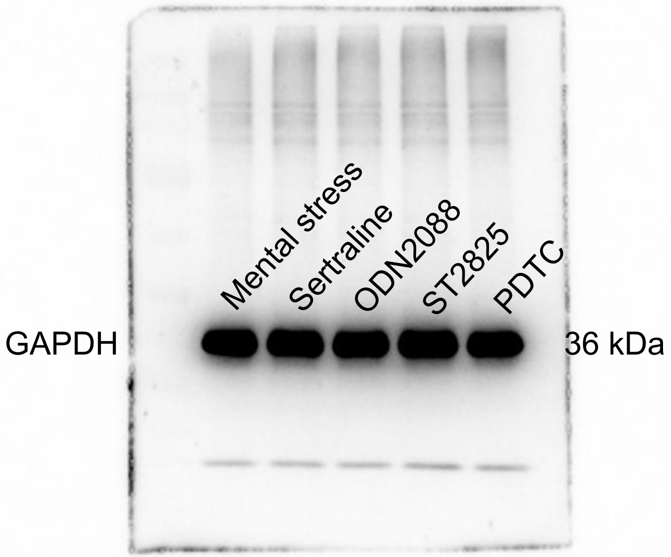
Mental stress
Sertraline
ODN2088
ST2825
PDTC
GAPDH
36 kDa
