# Supplementary material for: TLR9/MyD88/NF-κB signaling mediates mental stress-induced exacerbation of psoriasis through immune dysregulation in a mouse model
Source: PLoS One. 2026 Mar 6;21(3):e0344474. doi: 10.1371/journal.pone.0344474 (PMC12965552; doi:10.1371/journal.pone.0344474)
Supplement: Attachment — Submitted filename: Response to Reviewers.docx [file pone.0344474.s002.docx]

Dear Editor and Reviewers:

Thank you very much for your letter and for the reviewers’ comments concerning our manuscript entitled **"TLR9/MyD88/NF-κB signaling mediates mental stress-induced exacerbation of psoriasis through immune dysregulation in a mouse model" (ID: PONE-D-25-36503).** The main corrections in the paper and the responds to the reviewers’ comments are as follows:

**Responds to** **editor**

When submitting your revision, we need you to address these additional requirements.

1. Please ensure that your manuscript meets PLOS ONE's style requirements, including those for file naming. The PLOS ONE style templates can be found at https://journals.plos.org/plosone/s/file?id=wjVg/PLOSOne_formatting_sample_main_body.pdf and https://journals.plos.org/plosone/s/file?id=ba62/PLOSOne_formatting_sample_title_authors_affiliations.pdf

Response: Thanks for your advice. We have revised the manuscript and ensured that it meets PLOS ONE's style requirements.

2. Thank you for stating the following in the Acknowledgments Section of your manuscript:

“This study was supported by National Natural Science Foundation of China (No. 82260283) and The Science and Technology Plan Project of Provincial Health Commission in 2025 (No. 202510823).”

We note that you have provided funding information that is not currently declared in your Funding Statement. However, funding information should not appear in the Acknowledgments section or other areas of your manuscript. We will only publish funding information present in the Funding Statement section of the online submission form.

Please remove any funding-related text from the manuscript and let us know how you would like to update your Funding Statement. Currently, your Funding Statement reads as follows:

“The author(s) received no specific funding for this work.”

Please include your amended statements within your cover letter; we will change the online submission form on your behalf.

Response: Thanks for your advice. We feel sorry for our carelessness. In fact, the Acknowledgments Section should state “Not applicable”, while the statement “This study was supported by National Natural Science Foundation of China (No. 82260283) and The Science and Technology Plan Project of Provincial Health Commission in 2025 (No. 202510823)” should appear in the Funding Statement Section.

According to the requirements, we have amended the statements within cover letter.

3. Thank you for stating the following financial disclosure:

“The author(s) received no specific funding for this work.”

At this time, please address the following queries:

a) Please clarify the sources of funding (financial or material support) for your study. List the grants or organizations that supported your study, including funding received from your institution.

b) State what role the funders took in the study. If the funders had no role in your study, please state: “The funders had no role in study design, data collection and analysis, decision to publish, or preparation of the manuscript.”

c) If any authors received a salary from any of your funders, please state which authors and which funders.

d) If you did not receive any funding for this study, please state: “The authors received no specific funding for this work.”

Please include your amended statements within your cover letter; we will change the online submission form on your behalf.

Response: Thanks for your advice. As mentioned in comment 2, we have amended the statements within cover letter.

4. Thank you for stating the following financial disclosure:

“The author(s) received no specific funding for this work.”

Please state what role the funders took in the study. If the funders had no role, please state: "The funders had no role in study design, data collection and analysis, decision to publish, or preparation of the manuscript."

If this statement is not correct you must amend it as needed.

Please include this amended Role of Funder statement in your cover letter; we will change the online submission form on your behalf.

Response: Thanks for your advice. As mentioned in comment 2, we have amended the statements within cover letter.

5. We note that your Data Availability Statement is currently as follows: “All relevant data are within the manuscript and its Supporting Information files.”

Please confirm at this time whether or not your submission contains all raw data required to replicate the results of your study. Authors must share the “minimal data set” for their submission. PLOS defines the minimal data set to consist of the data required to replicate all study findings reported in the article, as well as related metadata and methods (https://journals.plos.org/plosone/s/data-availability#loc-minimal-data-set-definition).

For example, authors should submit the following data:

- The values behind the means, standard deviations and other measures reported;

- The values used to build graphs;

- The points extracted from images for analysis.

Authors do not need to submit their entire data set if only a portion of the data was used in the reported study.

If your submission does not contain these data, please either upload them as Supporting Information files or deposit them to a stable, public repository and provide us with the relevant URLs, DOIs, or accession numbers. For a list of recommended repositories, please see https://journals.plos.org/plosone/s/recommended-repositories.

If there are ethical or legal restrictions on sharing a de-identified data set, please explain them in detail (e.g., data contain potentially sensitive information, data are owned by a third-party organization, etc.) and who has imposed them (e.g., an ethics committee). Please also provide contact information for a data access committee, ethics committee, or other institutional body to which data requests may be sent. If data are owned by a third party, please indicate how others may request data access.

Response: Thanks for your advice. All raw data of this study have been uploaded in Zenodo. Additionally, we have revised the Data Availability Statement as “The datasets generated and/or analyzed during the current study are available in Zenodo, the open-access repository operated by CERN, [https://zenodo.org/records/18069564?token=eyJhbGciOiJIUzUxMiJ9.eyJpZCI6ImZjYzdkNmNlLTFhZjktNDE3ZS05YzdiLTI2NjhmM2U4MWY1OSIsImRhdGEiOnt9LCJyYW5kb20iOiJmYTBjMmIyZDAyNjk2Nzg0NTgxNGJjODRhMTExNDFkZCJ9.HeVpzwu5n4OdiSw3toFMjTZBCfdZc_uQMNAjmFJ4yxSigNemmWOl5bSr0zyMPHrk74bYpXBmdUajlgSLbbN7-w]”

6. PLOS ONE now requires that authors provide the original uncropped and unadjusted images underlying all blot or gel results reported in a submission’s figures or Supporting Information files. This policy and the journal’s other requirements for blot/gel reporting and figure preparation are described in detail at https://journals.plos.org/plosone/s/figures#loc-blot-and-gel-reporting-requirements and https://journals.plos.org/plosone/s/figures#loc-preparing-figures-from-image-files. When you submit your revised manuscript, please ensure that your figures adhere fully to these guidelines and provide the original underlying images for all blot or gel data reported in your submission. See the following link for instructions on providing the original image data: https://journals.plos.org/plosone/s/figures#loc-original-images-for-blots-and-gels.

In your cover letter, please note whether your blot/gel image data are in Supporting Information or posted at a public data repository, provide the repository URL if relevant, and provide specific details as to which raw blot/gel images, if any, are not available. Email us at plosone@plos.org if you have any questions.

Response: Thanks for your advice. The original uncropped and unadjusted gels of this study have been uploaded in Zenodo, the open-access repository operated by CERN, [https://zenodo.org/records/18069564?token=eyJhbGciOiJIUzUxMiJ9.eyJpZCI6ImZjYzdkNmNlLTFhZjktNDE3ZS05YzdiLTI2NjhmM2U4MWY1OSIsImRhdGEiOnt9LCJyYW5kb20iOiJmYTBjMmIyZDAyNjk2Nzg0NTgxNGJjODRhMTExNDFkZCJ9.HeVpzwu5n4OdiSw3toFMjTZBCfdZc_uQMNAjmFJ4yxSigNemmWOl5bSr0zyMPHrk74bYpXBmdUajlgSLbbN7-w]. According to the requirements, we have clarified this in cover letter.

7. If the reviewer comments include a recommendation to cite specific previously published works, please review and evaluate these publications to determine whether they are relevant and should be cited. There is no requirement to cite these works unless the editor has indicated otherwise.

Response: Thanks for your advice. As you mentioned above, the comment 7 of reviewer 2 include a recommendation to cite three previously published works (PMID: 35032680, PMID: 38453637, and PMID: 37573970). After a careful review of these three references, we summarize their core themes as follows:

-PMID: 35032680: Lycopene attenuates oxidative stress, inflammation, and apoptosis by modulating Nrf2/NF-κB balance in sulfamethoxazole-induced neurotoxicity in grass carp (Ctenopharyngodon Idella)

This study investigates the protective effect of lycopene against sulfamethoxazole-induced neurotoxicity in grass carp (Ctenopharyngodon idella), demonstrating that lycopene effectively alleviates oxidative stress, inflammation and apoptosis in the fish’s nervous system by regulating the balance of the Nrf2 and NF-κB signaling pathways, thus providing a potential strategy to mitigate the adverse impacts of sulfamethoxazole exposure in aquaculture.

-PMID: 38453637: Endoplasmic reticulum stress-induced NLRP3 inflammasome activation as a novel mechanism of polystyrene microplastics (PS-MPs)-induced pulmonary inflammation in chickens

This study identifies that endoplasmic reticulum stress-triggered NLRP3 inflammasome activation serves as a novel mechanism underlying polystyrene microplastics (PS-MPs)-induced pulmonary inflammation in chickens, revealing that PS-MPs exposure causes pulmonary pathological damage, initiates endoplasmic reticulum stress, and further activates NLRP3 inflammasome to induce pyroptosis and inflammatory responses via relevant signaling pathways including NF-κB, thus providing new insights into the pulmonary toxicological mechanisms of microplastics.

- PMID: 37573970: Evodiamine alleviates DEHP-induced hepatocyte pyroptosis, necroptosis and immunosuppression in grass carp through ROS-regulated TLR4 / MyD88 / NF-κB pathway

This study investigates the protective effect of evodiamine against DEHP-induced hepatotoxicity in grass carp, demonstrating that DEHP activates the ROS-regulated TLR4/MyD88/NF-κB pathway to trigger hepatocyte pyroptosis, necroptosis, and immunosuppression, while evodiamine alleviates these adverse effects by inhibiting ROS production, blocking pathway activation, and restoring hepatocellular immune function.

These three studies are not only irrelevant to our research in terms of disease types but also exhibit significant differences in species (mouse, fish, chicken), target organs (skin, liver, lung, nerve), and initiating stimuli (mental stress, pollutants, drugs). Therefore, after careful evaluation, we decided not to cite these three references in accordance with the editor’s requirements.

**Responds to the reviewers**

**Reviewer Comments**

1. Is the manuscript technically sound, and do the data support the conclusions?

The manuscript must describe a technically sound piece of scientific research with data that supports the conclusions. Experiments must have been conducted rigorously, with appropriate controls, replication, and sample sizes. The conclusions must be drawn appropriately based on the data presented.

Reviewer #1: Yes

Reviewer #2: Yes

Response: Thanks for your approval.

________________________________________

2. Has the statistical analysis been performed appropriately and rigorously?

Reviewer #1: Yes

Reviewer #2: Yes

Response: Thanks for your approval.

________________________________________

3. Have the authors made all data underlying the findings in their manuscript fully available?

The PLOS Data policy requires authors to make all data underlying the findings described in their manuscript fully available without restriction, with rare exception (please refer to the Data Availability Statement in the manuscript PDF file). The data should be provided as part of the manuscript or its supporting information, or deposited to a public repository. For example, in addition to summary statistics, the data points behind means, medians and variance measures should be available. If there are restrictions on publicly sharing data—e.g. participant privacy or use of data from a third party—those must be specified.

Reviewer #1: Yes

Reviewer #2: Yes

Response: Thanks for your approval.

________________________________________

4. Is the manuscript presented in an intelligible fashion and written in standard English?

PLOS ONE does not copyedit accepted manuscripts, so the language in submitted articles must be clear, correct, and unambiguous. Any typographical or grammatical errors should be corrected at revision, so please note any specific errors here.

Reviewer #1: Yes

Reviewer #2: Yes

Response: Thanks for your approval.

________________________________________

5. Review Comments to the Author

Please use the space provided to explain your answers to the questions above. You may also include additional comments for the author, including concerns about dual publication, research ethics, or publication ethics. (Please upload your review as an attachment if it exceeds 20,000 characters)

**Reviewer #1:**

In the manuscript titled “TLR9/MyD88/NF-κB signaling mediates mental stress-induced exacerbation of psoriasis through immune dysregulation in a mouse model”, this article mainly explores the mechanisms by which mental stress exacerbates human psoriasis. The results first showed that TLR9/MyD88/NF-κB pathway related molecules were significantly upregulated in peripheral blood mononuclear cells of psoriasis patients and psoriasis mouse models, while the expression of inflammatory factors was elevated. Subsequently, the article found for the first time that mental stress was shown to activate the TLR9/MyD88/NF - κ B pathway, enhance the production of inflammatory factors, increase the Th17/Treg ratio, and decrease the Treg/CD4+T ratio. The use of antipsychotic drugs or pathway inhibitors can inhibit this change. Overall, these findings provide valuable insights into the pathophysiological mechanisms underlying the progression of psoriasis, particularly elucidating the immune regulatory axis mediated by mental stress. But there are the following key issues that affect its publication value:

1. What is the relationship between TLR9/NF-κB pathway and Th17/Treg ratio, as well as Treg/CD4+T ratio? Explain in the introduction why these two ratios were chosen as detection indicators to ensure contextual coherence.

Response: Thanks for your advice. According to the requirements, we have explained the queries mentioned in the above comments. The detailed descriptions are in the second and third paragraph of Introduction section and shown as follows (red):

Mental stress plays a key role in the development of several skin diseases. The stigma associated with these conditions may exacerbate psychological burden, thereby creating a vicious cycle that further contributes to the development of skin diseases (18). The prevalence of psoriasis is notably higher among individuals who experience major stressful events (19). Furthermore, mental stress is the second most important factor contributing to psoriasis exacerbation after seasonal changes (20). Recent studies have suggested that mental stress modulates skin immune imbalance to promote psoriasis progression, which is associated with the Toll-like receptor (TLR) pathway (18, 21), and regulates the expression of cytokines such as IL-17 and IL-23 (22-26). Th17 cells exacerbate skin inflammatory infiltration by secreting pro-inflammatory cytokines such as IL-17 and IL-23, whereas regulatory T (Treg) cells maintain immune homeostasis through the secretion of anti-inflammatory cytokines including IL-10 and transforming growth factor-β (TGF-β). The dynamic balance between these two cell subsets, reflected by the Th17/Treg ratio, directly regulates the inflammatory severity and disease progression of psoriasis [PMID: 36444619]. Clinical studies have confirmed that the Th17/Treg ratio is significantly elevated in the peripheral blood and lesional tissues of psoriasis patients [PMID: 37596509]. Therefore, this ratio is regarded as a core indicator for evaluating immune dysregulation in psoriasis. Meanwhile, as a critical subset of CD4⁺T cells, Treg/CD4⁺T ratio directly reflects the integrity of immune regulatory function. Under the pathological conditions of psoriasis, the proliferation, activation, and immunosuppressive capacity of Treg cells are impaired, leading to a decreased Treg/CD4⁺T ratio. This reduction further weakens the inhibitory effect on aberrant inflammatory responses [PMID: 37812989]. TLRs are extensively expressed on immune cells and cellular membranes (27). These receptors are activated through distinct signaling pathways, primarily mediated by either myeloid differentiation primary response 88 (MyD88) or TIR-domain-containing adaptor-inducing interferon-beta. These signaling cascades ultimately lead to enhanced production and secretion of inflammatory cytokines, thereby modulating the immune response and maintaining organismal homeostasis (28). Furthermore, NF-κB plays an important role as a downstream signal of TLRs (29, 30).

Emerging evidence has demonstrated that mental stress modulates the expression of local TLRs in the cerebral cortex, triggering disruptions in regional immune homeostasis through alterations in cytokine profiles and immune cell population dynamics (31). These neuroimmune changes are closely associated with the development of psychiatric manifestations, including depression, anxiety, and suicidal behaviors (32, 33). As a crucial member of the TLR family, the abnormal expression of TLR9 is observed in various stress-related disorders. For example, Hung et al. (34) identified elevated levels of TLR9 in peripheral blood mononuclear cells (PBMCs) isolated from patients with major depressive disorder. Furthermore, Zhang et al. (35) provided experimental evidence indicating that exposure to chronic stress leads to an upregulation of TLR9 expression in murine blood samples, accompanied by an increase in TGF-β production. Collectively, these findings suggest that TLR9 serves as a crucial mediator of mental stress-induced neuroimmune dysregulation, and its altered expression patterns can significantly influence downstream cytokine signaling pathways within the nervous system. Notably, as a crucial hub bridging innate and adaptive immunity, the TLR9/MyD88/NF-κB signaling pathway modulates the Th17/Treg balance through multiple mechanisms. On one hand, TLR9/MyD88-mediated NF-κB pathway activation can promote dendritic cells to secrete cytokines such as IL-23 and IL-1β, which specifically induce the differentiation of naive CD4⁺T cells into Th17 cells [PMID: 41373468]. On the other hand, sustained NF-κB activation suppresses the expression of the Foxp3 transcription factor in Treg cells, thereby impairing the stability and immunosuppressive function of Treg cells [PMID: 35672519]. Accumulating evidence has verified that in autoimmune disease models, inhibitors targeting the TLR9/NF-κB pathway can remarkably reduce the Th17/Treg ratio while elevating the Treg/CD4⁺T ratio, ultimately alleviating inflammatory responses [PMID: 39744924]. However, the interaction between mental stress and TLR9/MyD88/NF-κB signaling pathwayTLR9 as well as the possible regulatory mechanism during the progression of psoriasis are still rarely reported.

2. Phosphorylated NF-κB P65 also plays a very important role in the inflammatory process, especially in this inflammatory axis. Why not study p-NF-κB P65?

Response: Thanks for your advice. Initially, this study focused on the activation status of the TLR9/MyD88/NF-κB pathway. By detecting the total protein and mRNA levels of NF-κB p65, we verified the activating effect of mental stress on this pathway. Given that the preliminary focus of the study was to clarify whether the pathway is activated and its association with immune imbalance, we prioritized the detection of core pathway molecules, downstream inflammatory cytokines, and immune cell subsets, while the in-depth investigation of phosphorylated NF-κB p65 (p-NF-κB p65) was temporarily postponed.

We fully concur with the reviewers’ viewpoint that the activation of the NF-κB pathway is dependent on the phosphorylation of NF-κB p65. The phosphorylated NF-κB p65 translocates from the cytoplasm to the nucleus, thereby initiating the transcription of downstream target genes, which represents a pivotal step in inflammatory signal transduction. Therefore, we supplemented western blotting and immunofluorescence assay for the measurement of p-NF-κB p65 expression. The images are in the revised figure 2G, 4A-B, and 6F.

3. The changes in NF-κB protein are generally not simply an increase or decrease in quantity. The process of inflammatory activation is accompanied by complex nuclear translocation and other processes. Why has no further exploration been conducted? Please provide a detailed explanation

Response: Thanks for your advice. As mentioned in comment 2, we performed immunofluorescence assay (the revised figure 2G, 4B and 6F) to detect the fluorescence intensity of p-NF-κB p65. The fluorescent signal of p-NF-κB p65 was specifically enhanced in the nucleus, accompanied by a relative attenuation of its signal in the cytoplasm. These results indirectly indicated the nuclear translocation of NF-κB p65.

4. The changes in the levels of inflammatory axis proteins and mRNA are very significant, and if validated from the perspective of cellular localization, it can further increase credibility. For example, immunofluorescence

Response: Thanks for your advice. According to the requirements, immunofluorescence assay was performed to assess the fluorescence intensity of IL-1β, IL-6, IL-17 and IL-23. The images are in the revised figure 2I, 4D, and 6H.

5. Line151 Please provide references for Internationally recognized method of solitary stress

Response: Thanks for your advice. According to the requirements, two references were provided as follows:

For the induction of mental stress, the internationally recognized method of solitary stress was employed in this study with slight modifications [PMID: 33488368, 36440892].

6. Interpret the results of the flow cytometry and how to determine the changes in Th17/Treg and Treg/CD4+T ratios based on the images of the flow cytometry results

Response: Thanks for your advice. Taking Figure 3A as an example, the CD4⁺T cell population was gated in the scatter plot (the area enclosed by the red box in the plot). Within the pre-gated CD4⁺T cell population, Treg cells were identified as the CD25⁺FOXP3⁺ subset (located in the upper right [UR] quadrant). Therefore, Treg/CD4⁺T ratio was calculated as (Treg cell population/CD4⁺T cell population) × 100%, and the results are presented in the adjacent bar graph.

We have clarified this in Results section.

7. Although the results of HE staining are obvious, I believe that adding a comparison of gross photographs of mouse skin would be more intuitive and persuasive.

Response: Thanks for your advice. According to the requirements, the photographs of mouse skin in the control, model and mental stress groups were provided in the revised figure 2A.

8. The current manuscript is recommended to be polished by professional language editing service personnel.

Response: Thanks for your advice. Our manuscript has been re-edited by a professional language editing service according to the requirements, and a “CERTIFICATE OF EDITING” has been uploaded.

Summary

This article needs to further improve the overall experimental logic and specific details.

Response: Thanks for your advice. We have supplemented additional experiments and provided more details in the revised manuscript according to the requirements, and hope that these modifications meet the requirements of the reviewers.

**Reviewer #2:**

This study investigates the mechanism by which psychological stress exacerbates psoriasis via the TLR9/MyD88/NF-κB signaling pathway. Utilizing both patient samples and a mouse model, the authors demonstrate that mental stress activates this pathway, promotes the release of inflammatory factors, and induces an imbalance in the Th17/Treg cell ratio. Inhibition of this pathway using pharmacological inhibitors and gene knockdown techniques alleviated the inflammatory response, improved immune balance, and mitigated skin lesions. The research reveals a novel mechanism through which mental stress aggravates psoriasis via immune dysregulation, offering a potential therapeutic target. The manuscript is recommended for publication after minor revisions.

Specific points for consideration:

1. In Figure 1, the data appear to show considerable variability among replicates. Furthermore, the selection of the Y-axis scale in panel G seems inappropriate and may misrepresent the data.

Response: Thanks for your advice. We would like to clarify that this variability is biologically reasonable and consistent with the characteristics of clinical samples (PBMCs from human subjects):

Intrinsic variability of clinical samples: PBMCs were isolated from independent human individuals (both healthy controls and psoriasis patients). Due to inherent differences in genetic background, disease severity, and baseline immune status among individual subjects, inter-individual variability in molecular/cytokine expression is a common and well-recognized feature in clinical immunology studies. This variability is reflective of the physiological heterogeneity in the human population, rather than experimental inconsistency.

Additionally, rigorous experimental and statistical design can ensure the reliability of the results. All samples were processed using standardized protocols (e.g., uniform PBMC isolation and consistent RNA extraction/cytokine detection procedures) to minimize technical variability.

In summary, the observed variability is biologically meaningful (reflecting human inter-individual heterogeneity) and does not compromise the validity of our conclusions, as supported by rigorous experimental procedures and significant statistical differences between groups.

Additionally, according to the requirements, the Y-axis scale in panel G was revised.

2. For the histological images presented in Figure 2A and Figure 6B, it is essential to include scale bars to provide context for the magnification.

Response: Thanks for your advice. According to requirements, we added scale bar (50 μm) for histological images (HE staining).

3. The description of the "single-housing stress" model should be elaborated. Providing behavioral data validating the effectiveness of this stress paradigm is highly recommended.

Response: Thanks for your advice. According to the requirements, behavioral tests including sucrose preference test and tail suspension test were provided. The images are in the revised figure 2B. Additionally, we also provided more details for the description of the "single-housing stress" model. The detailed descriptions in Materials and Methods section are as follows:

For the induction of mental stress, the internationally recognized method of solitary stress was employed in this study with slight modifications [PMID: 33488368, 36440892]. Detailed procedures were as follows: specialized solitary cages (12 cm × 10 cm × 15 cm) were used, which provided only enough space for mice to move comfortably (i.e., allow free turning but restrict excessive locomotion) to induce mild physical constraint without causing physical injury. Each solitary cage was placed inside a larger transparent cage (40 cm × 30 cm × 25 cm), and the cage was covered with a light-impermeable black hood to create a dark environment, minimizing visual stimulation and simulating an isolated, unfamiliar context. No food or water was placed in the solitary cages during the stress period to avoid confounding effects of nutrient intake on stress responses. The solitary stress was initiated one week after mice adaptation to the animal facility and continued for 10 consecutive weeks. To avoid habituation, stress exposure was conducted from 7:00 to 9:00 a.m. three times per week (Monday, Wednesday, and Friday), with each session lasting 2 h. Immediately after each 2-hour stress session, mice were returned to their original colony cages (30 cm × 20 cm × 15 cm) with ad libitum access to food and water. The colony cages were maintained in a standardized environment (22±1°C, 50±5% relative humidity, 12-h light/dark cycle), ensuring consistent environmental cues except for the stress intervention.

4. Certain sections of the Materials and Methods could be condensed for brevity and clarity.

Response: Thanks for your advice. According to the requirements, we have split certain content of the Materials and Methods section and removed some redundant descriptions.

5. In Figure 4A, the bar representing the data for the control group appears to be missing. Please clarify or correct this representation.

Response: Thanks for your advice. We would like to clarify that the omission of the control group bar in Figure 4A is intentional and scientifically reasonable, based on the specific research focus and experimental design of this study, as elaborated below:

Figure 4A is designed to evaluate the regulatory effects of TLR9/MyD88/NF-κB pathway inhibitors (ODN2088, ST2825, PDTC) and the antidepressant Sertraline (as a positive control) on the TLR9/MyD88/NF-κB signaling pathway in mental stress-exposed psoriatic mice. The core comparison is between the "model + mental stress group" (the baseline group for intervention efficacy) and the respective intervention groups, rather than between the control group and intervention groups.

In fact, the control group (without psoriasis induction or mental stress) exhibits minimal activation of the TLR9/MyD88/NF-κB pathway, which has been clarified in figure 2. This low baseline expression is a well-established background and does not contribute to evaluating the intervention effects on stress-aggravated psoriasis. Including the control group bar would introduce irrelevant baseline data, increasing visual complexity without enhancing the clarity of the key comparison. Additionally, the entire Figure 4 focuses on the "inflammation-attenuating effects of interventions in mental stress-treated psoriatic mice". All corresponding analyses (e.g., inflammatory cytokine levels in the revised Figure 4C) use the "model + mental stress group" as the reference. Omitting the normal control group in Figure 4A maintains consistency in the comparative framework across the figure, avoiding misleading interpretations.

In summary, the exclusion of the control group bar in Figure 4A is a deliberate choice to prioritize the clarity of intervention efficacy comparisons, which aligns with the research focus of this figure and the overall experimental design. This representation does not compromise the scientific rigor or completeness of the data, as the necessary baseline control information is provided elsewhere in the manuscript.

6. For the H&E staining images, using boxes or arrows to highlight specific pathological features would greatly enhance clarity for the reader.

Response: Thanks for your advice. According to the requirements, we used arrows to highlight specific pathological features as follows: black arrows indicate the epidermal layer, red arrows mark the dermal layer, and blue arrows identify the hair follicles. We have clarified this in Results section.

7. For references on inflammation and immune system discussions, PMID: 35032680, PMID: 38453637, and PMID: 37573970.

Response: Thanks for your advice. In fact, the comment 7 of editor include a specifical statement as follows:

If the reviewer comments include a recommendation to cite specific previously published works, please review and evaluate these publications to determine whether they are relevant and should be cited. There is no requirement to cite these works unless the editor has indicated otherwise.

This was specifically requested by the editor in the revision comments.

After careful evaluation, we believe that these three studies are not only irrelevant to our research in terms of disease types but also exhibit significant differences in species (mouse, fish, chicken), target organs (skin, liver, lung, nerve), and initiating stimuli (mental stress, pollutants, drugs). Therefore, we decided not to cite these three references in accordance with the editor’s requirements. Thanks for your scientific suggestion again.
