# Supplementary material for: TLR9/MyD88/NF-κB signaling mediates mental stress-induced exacerbation of psoriasis through immune dysregulation in a mouse model
Source: PLoS One. 2026 Mar 6;21(3):e0344474. doi: 10.1371/journal.pone.0344474 (PMC12965552; doi:10.1371/journal.pone.0344474)
Supplement: Attachment — Submitted filename: renamed_7c3d5.docx [file pone.0344474.s003.docx]

This study reveals that TLR9/MyD88/NF-κB pathway related molecules were significantly upregulated in peripheral blood mononuclear cells of psoriasis patients and psoriasis mouse models, while the expression of inflammatory factors was elevated. Subsequently, the article found for the first time that mental stress was shown to activate the TLR9/MyD88/NF - κ B pathway, enhance the production of inflammatory factors, increase the Th17/Treg ratio, and decrease the Treg/CD4+T ratio. The use of antipsychotic drugs or pathway inhibitors can inhibit this change. There are no further questions that need to be discussed.
